# Erythromycin mediates co-flocculation between cyanobacterium *Synechocystis* sp. PCC 6803 and filamentous fungi in liquid cultivation without organic compounds

**DOI:** 10.1038/s41598-024-60016-7

**Published:** 2024-04-26

**Authors:** Panutchaya Pichaiyotinkul, Jidapa Leksingto, Nannaphat Sukkasam, Pichaya In-na, Aran Incharoensakdi, Tanakarn Monshupanee

**Affiliations:** 1 Department of Biochemistry, Faculty of Science, Chulalongkorn University, Bangkok, 10330 Thailand; 2 Research Unit on Sustainable Algal Cultivation and Applications, Chulalongkorn University, Bangkok, 10330 Thailand; 3 Department of Chemical Technology, Faculty of Science, Chulalongkorn University, Bangkok, 10330 Thailand; 4 Academy of Science, Royal Society of Thailand, Bangkok, 10300 Thailand

**Keywords:** Antibiotic, Cyanobacteria, Flocculation, Gene expression, Cell harvest, Stress, Biotechnology, Microbiology, Applied microbiology

## Abstract

Photoautotrophic cyanobacteria assimilate the greenhouse gas carbon dioxide as their sole carbon source for producing useful bioproducts. However, harvesting the cells from their liquid media is a major bottleneck in the process. Thus, an easy-to-harvest method, such as auto-flocculation, is desirable. Here, we found that cyanobacterium *Synechocystis* sp. PCC 6803 co-flocculated with a natural fungal contamination in the presence of the antibiotic erythromycin (EM) but not without EM. The fungi in the co-flocculated biomass were isolated and found to consist of five species with the filamentous *Purpureocillium lilacinum* and *Aspergillus protuberus* making up 71% of the overall fungal population. The optimal co-cultivation for flocculation was an initial 5 mg (fresh weight) of fungi, an initial cell density of *Synechocystis* of 0.2 OD_730_, 10 µM EM, and 14 days of cultivation in 100 mL of BG11 medium with no organic compound. This yielded 248 ± 28 mg/L of the *Synechocystis*-fungi flocculated biomass from 560 ± 35 mg/L of total biomass, a 44 ± 2% biomass flocculation efficiency. Furthermore, the EM treated *Synechocystis* cells in the *Synechocystis*-fungi flocculate had a normal cell color and morphology, while those in the axenic suspension exhibited strong chlorosis. Thus, the occurrence of the *Synechocystis*-fungi flocculation was mediated by EM, and the co-flocculation with the fungi protected *Synechocystis* against the development of chlorosis. Transcriptomic analysis suggested that the EM-mediated co-flocculation was a result of down-regulation of the minor pilin genes and up-regulation of several genes including the chaperone gene for pilin regulation, the S-layer protein genes, the exopolysaccharide-polymerization gene, and the genes for signaling proteins involved in cell attachment and abiotic-stress responses. The CuSO_4_ stress can also mediate *Synechocystis*-fungi flocculation but at a lower flocculation efficiency than that caused by EM. The EM treatment may be applied in the co-culture between other cyanobacteria and fungi to mediate cell bio-flocculation.

## Introduction

*Synechocystis* sp. PCC 6803 (hereafter, *Synechocystis*) is one of best-studied cyanobacteria that produces useful bioproducts, such as polysaccharides, bioplastic polymers, lipids, hydrogen gas, and pigments^1–4^. The well-characterized metabolism and the available gene manipulation in *Synechocystis* enable rational metabolic engineering to produce industrial bioenergy and biochemical compounds^5–7^.

However, harvesting of the cells from the liquid growth medium is one of the main barriers for producing such products by *Synechocystis* and other cyanobacteria and algae^8,9^. To harvest cyanobacterial cells from liquid culture, physical processes (sedimentation, filtration, and centrifugation), and chemical flocculation have been used^9–12^, but these techniques have a high operation cost and excessive energy consumption^9,11,13^. Biological flocculation, which requires cell–cell attachment between cyanobacteria-cyanobacteria^14–17^, cyanobacteria-bacteria^15,18^, and cyanobacteria-fungi^12,19^, is a promising advantage because this approach does not require a high cost or extra energy^12,19^. The co-flocculation between cyanobacteria and fungi is interesting because of its high efficiency of biomass flocculation in a liquid culture^20–22^.

The co-flocculation of cyanobacteria-fungi has been previously described^20–22^. For *Synechocystis*, co-flocculated *Synechocystis*-*Aspergillus fumigatus* and *Synechocystis*-*A. oryzae* were formed by adding a substantial amount of pre-grown fungal pellets to the *Synechocystis* culture^19,21^. The biological benefit for the co-flocculation between cyanobacteria-fungi and between the microalgae-fungi in lichens has been known for nutrient exchange^20,23^. However, knowledge on the cellular mechanisms of flocculation between cyanobacteria and fungi is still limited^24^. Pilin, exopolysaccharides (EPS), and S-layer protein on the cell surface of cyanobacteria have all been reported to play a role in cell–cell attachment of cyanobacterial auto-flocculation^25–27^. In cyanobacteria-fungi co-flocculation, previous studies described that cell surface attachment is mediated by the electrostatic interaction between the negative charges of EPS on the cyanobacterial cell surface and the positive charges of saccharides on the cell surface of fungal hypha^21,28^. The S-layer protein is also required for cell–cell attachment, since the *Synechocystis* mutant lacking S-layer protein has been shown to have a significantly reduced biofilm formation^25^. In addition, pilins are known to be involved in the motility and cell adhesion of bacteria^29^ and cyanobacteria^30–32^.

In nature, various microorganisms produce antibiotics that inhibit bacterial growth or kill bacteria^33,34^. Bacteria can alleviate growth inhibition by antibiotic by forming a biofilm to protect the cells from antibiotic exposure and absorption^35,36^. The consortium cultivation between bacteria and eukaryotic green algae helps to reduce exposure to antibiotics in liquid culture^37,38^. However, the consortium between cyanobacteria and other organisms for alleviating antibiotic activity has yet to be studied. In cyanobacterium *Synechocystis*, among various antibiotics that inhibit cell growth^39^, erythromycin (EM) is of interest because it exhibits bactericidal activity to *Synechocystis*^39,40^ via interfering with bacterial protein synthesis by blocking a nascent peptide exit tunnel of the large ribosomal subunit^41^.

In this study, *Synechocystis-*fungi flocculation was observed in a *Synechocystis* culture with a natural fungal contamination in the presence of EM. This study hypothesized that *Synechocystis* co-flocculates with the fungi under the presence of EM. To prove this, the fungi were then isolated on agar medium, and the isolated fungi and *Synechocystis* were co-cultivated in a liquid medium in the presence or absence of EM. This study also aims to identify: (i) the fungal population present in the co-flocculated biomass; (ii) the optimal inoculation amounts of *Synechocystis* and fungi and the optimal initial EM concentration for the maximal biomass flocculation; (iii) potential *Synechocystis* genes responsible for *Synechocystis-*fungi flocculation. This study also confirmed this bioflocculation formation by illustrating the cell morphology of the fungi and *Synechocystis* in the flocculated biomass using bright-field and fluorescent microscopy.

## Methods

### Strain and culture medium

The cyanobacterium *Synechocystis* sp. PCC 6803 was obtained from Institute Pasteur, France. *Synechocystis* was cultured in the standard BG11 medium (hereafter, BG11)^42^ supplemented with 20 mM [4-(2-hydroxyethyl)-1-piperazineethanesulfonic acid]–NaOH (pH 7.5) but without sodium citrate and with the ferric ammonium citrate being replaced with ferric chloride. Therefore, this BG11 medium does not contain any organic compounds^43^ in order to limit the growth of the fungi. The fungi were first obtained from a natural contamination in a *Synechocystis* culture. Subsequently, the fungi were isolated by streaking onto BG11 medium agar plates containing 1.5% (w/v) bacto agar (Himedia, India), which is the sole carbon source of the fungi. For fungal growth, the BG11 agar plates were incubated at 37 °C for 2–3 days in the dark. The contaminated fungi were re-streaked four times.

### *Synechocystis* axenic cultivation, co-cultivation of *Synechocystis* and fungi, and cell harvesting

All cultivations were operated in 100 mL BG11 liquid medium under continuous cool white fluorescent light (50 μmol m^−2^ s^−1^) at 28 °C with constant shaking at 160 rpm, and an atmospheric carbon dioxide (CO_2_) concentration [0.04% (v/v)] was supplied. The EM was added to the BG11 medium when required. For *Synechocystis* axenic cultivation, only *Synechocystis* was cultivated. For the co-cultivation of *Synechocystis* and fungi, *Synechocystis* and fresh fungal biomass (harvested from the agar plate) were mixed and cultivated for 7–14 days. Flocculated biomass was harvested from the liquid culture using gravitational filtration through an aluminum filter with a pore size of 0.05 mm. The remaining biomass in the liquid medium after filtration was collected using centrifugation. The flocculated and suspended biomass were dried at 60 °C until at a constant weight was achieved.

Calculation of % flocculated biomass and % suspended biomass with respect to total biomass

$$\begin{aligned} & {\text{\% }}\,{\text{Flocculated}}\,{\text{biomass}} \\ & \quad = \frac{{Flocculated\,biomass\,{\text{in}}\,{\text{mg}}/{\text{L}}}}{{\left( {Flocculated\,biomass\,{\text{in}}\,{\text{mg}}/{\text{L}}} \right){ } + { }\left( {Suspended\,biomass\,{\text{in}}\,{\text{mg}}/{\text{L}}} \right)}} \times 100 \\ & {\text{\% }}\,{\text{Suspended}}\,{\text{biomass}} \\ & \quad = \frac{{Suspended\,biomass\,{\text{in}}\,{\text{mg}}/{\text{L}}}}{{\left( {Flocculated\,biomass\,{\text{in}}\,{\text{mg}}/{\text{L}}} \right){ } + { }\left( {Suspended\,biomass\,{\text{in}}\,{\text{mg}}/{\text{L}}} \right)}} \times 100 \\ \end{aligned}$$

### Microscopic examination of the flocculated cells

Each *Synechocystis*-fungi flocculated biomass was visualized under light microscopy (BX51; Olympus Corporation, Tokyo, Japan). For observation of the overall cell morphology, cells were observed under bright-field mode using a standard white light. For examination of chlorophyll in *Synechocystis* cells, the autofluorescence of chlorophyll (seen as red fluorescent light) was detected using an excitation filter (530–535 nm) and an emission filter (> 580 nm).

### Transcriptomics analysis

The transcriptomes of *Synechocystis* from three different groups were analyzed (Table 2). Each group was performed using biomass from three independent cultures. For RNA extraction, fresh cells were quickly frozen with liquid nitrogen, milled with glass beads (150–212 μm, Cat. No. G9018, Sigma-Aldrich, Missouri, USA), and then TRIzol reagent (Life Technologies, Carlsbad, CA, USA) was used to extract RNA as described previously^44^. A RNase-free DNase kit (Qiagen, Hilden, Germany) was used to remove DNA contamination. The cDNA library was synthesized, and cDNA sequencing was performed at the OMICS SCIENCE center (Faculty of Science, Chulalongkorn University, Bangkok, Thailand) as previously described^44^. In brief, RNA strand-specific RNA-Seq libraries were created using the QIAseq FastSelect RNA removal and QIAseq stranded total RNA library preparation kits (QIAGEN). QIAseq FastSelect–rRNA HMR QIAseq FastSelect–5S/16S/23S-rRNA removal solution (QIAGEN, USA) was used to remove ribosomal RNAs. AMPure XP beads (Beckman Coulter Genomic) were used to enrich the RNA sample following cDNA synthesis.

All cDNA libraries were analyzed for quality using an Agilent 2100 Bioanalyzer (Agilent) after indexing adapters were ligated to the cDNAs. The sequencing libraries were pooled in an equimolar amount after the cDNA libraries were quantified using a DeNovix fluorometer (DeNovix). Pair-end 125 nucleotides read sequencing on an Illumina HiSeq sequencer was done with cluster creation. The quality of the raw read data was examined using the FASTQC software. Fastp software was used to delete adapters and low-quality reads. Salmon software was used to compare high-quality reads to the reference genome sequence (*Synechocystis* sp. PCC 6803, accession number: ASM972v1). DESeq2 was used for the differential gene expression analysis (FDR 0.05, log_2_ fold change > 1.0). The detected transcripts from fungi were excluded and were not used for transcriptomic analysis. The transcriptome's raw read data were submitted to the Gene Expression Omnibus (GEO) repository, accession numbers: GSE244152, GSE256451, and GSE256452.

### Fungal population determination

Total DNA from *Synechocystis*-fungi flocculated biomass was purified using DNeasy PowerSoil Pro DNA Kit (Qiagen, USA). The PCR amplification of the fungal internal transcribed spacer (ITS) rDNA was conducted using the ITS1 and ITS2 primers (Illumina, USA), using 2X sparQ HiFi PCR master Mix (QuantaBio, USA). The PCR thermal cycling was 2 min at 98 °C, followed by 30 cycles of 98 °C for 20 s, 60 °C for 30 s, and 72 °C for 1 min, and then followed by a final 72 °C for 1 min. Following enrichment using sparQ Puremag Beads (QuantaBio, USA) and indexing with 5 µL of each Nextera XT index primer in a 50-µL PCR reaction, the ITS amplicons were processed with 8–10 cycles of the above PCR procedure. Cleansing, pooling, and diluting the final PCR products to a final loading concentration of 4 pM were performed. The 250-bp paired-end read sequencing and cluster formation were performed on an Illumina MiSeq at the OMICS SCIENCE center (Faculty of Science, Chulalongkorn University, Bangkok, Thailand).

## Results

### *Synechocystis*-fungi flocculation is mediated by EM

**Figure 1:**
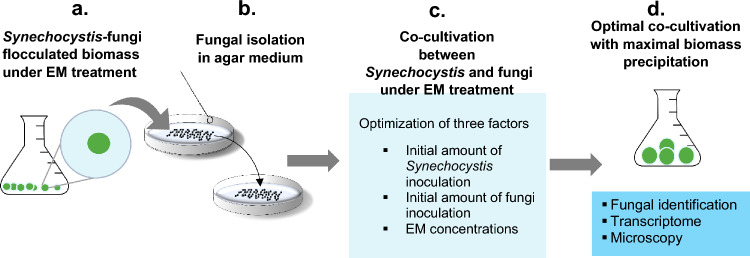
Outlines of the experimental procedures. (**a**) Flocculated biomass derived from the co-existence of *Synechocystis* and fungi was found in a *Synechocystis* culture under 10 μM EM treatment with a natural fungal contamination. (**b**) The flocculated biomass from (**a**) was streaked onto agar medium to isolate the fungi. (**c**) Co-cultivation between *Synechocystis* and the isolated fungi obtained from (**b**). This cultivation was optimized for the maximal level of flocculated biomass with respect to the three indicated factors. (**d**) The optimal co-cultivation yielding the maximal flocculated biomass was subjected to fungal classification by fungal ITS rDNA sequencing, *Synechocystis* transcriptomic analysis, and microscopic examination.

**Figure 2:**
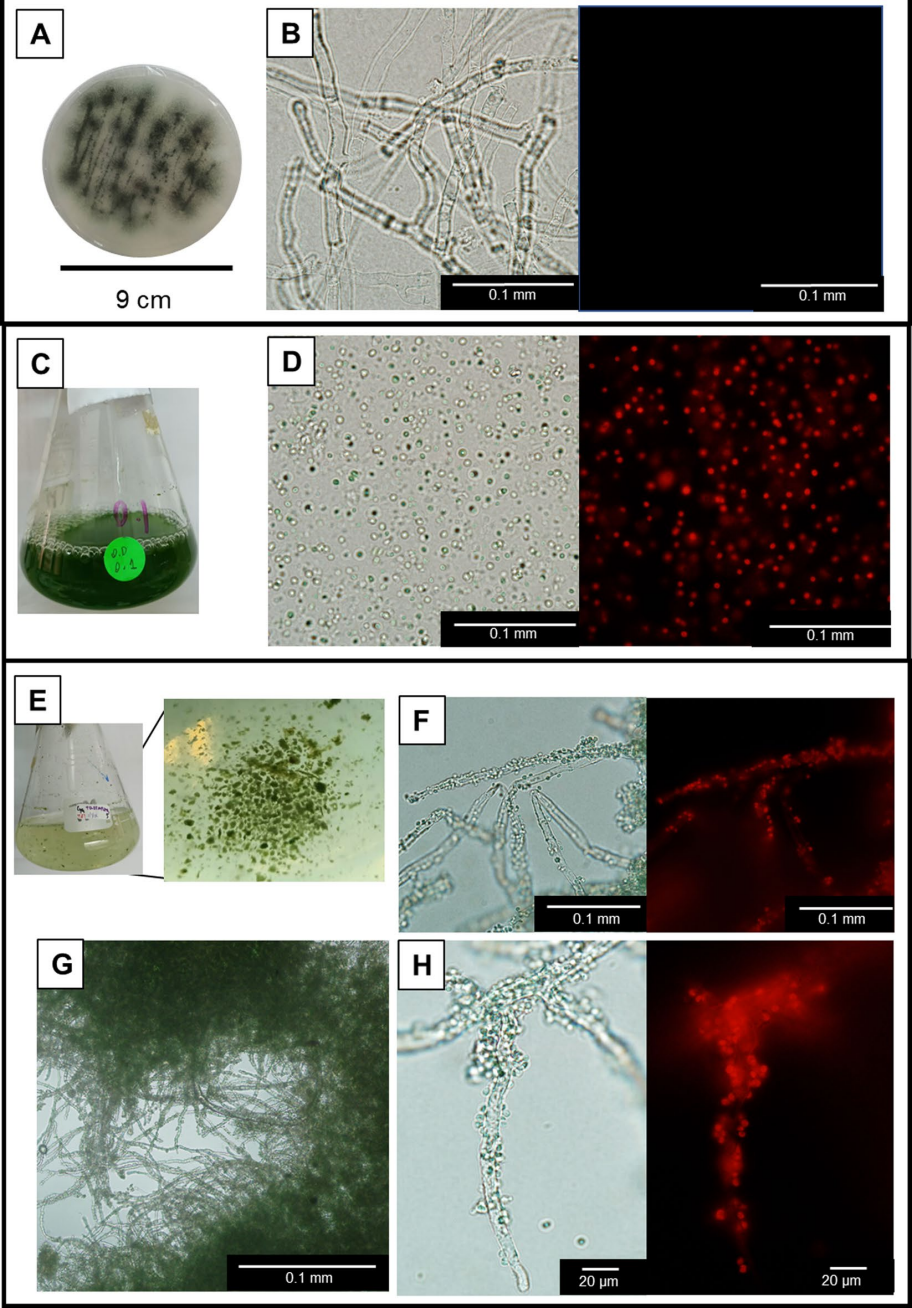
Culture and microscopic examination of *Synechocystis*-fungi flocculation. (**a**) Isolated fungi cultured on agar medium, as obtained from the procedure described in Fig. 1b. (**b**) Isolated fungi seen under bright-field (left) and fluorescent (right) microscopy. (**c**) Axenic *Synechocystis* cultured without EM. (**d**) Axenic *Synechocystis* from (**c**) as seen by bright-field microscopy (left) and fluorescent microscopy (right) showing the red light resulting from the auto-fluorescence of chlorophyll. (**e**) Co-cultivation between *Synechocystis* and the fungi with 10 µM EM showing *Synechocystis*-fungi flocculation. (**f**) *Synechocystis*-fungi flocculated biomass visualized by bright-field microscopy. (**g**, **h**) *Synechocystis*-fungi flocculated biomass as seen by bright-field microscopy (left) and fluorescent microscopy (right) showing the red light resulting from auto-fluorescence of *Synechocystis* chlorophyll.

A *Synechocystis*-fungi flocculated biomass was observed in a *Synechocystis* cultivation that was naturally contaminated with fungi upon the addition of EM. The fungi were subsequently isolated by re-streaking four times on an agar plate (Fig. 1). The isolated fungi exist in septate hyphae filaments (Fig. 2a,b).

To validate that the isolated fungi and *Synechocystis* co-flocculate in the presence of EM, the co-cultivation of *Synechocystis* and the isolated fungi was performed using an initial *Synechocystis* density of an optical density at 730 nm (OD_730_) of 0.2, a fungi inoculum of 5 mg, and 10 µM EM in 100 mL of the BG11 medium without any organic compounds. Under this condition, a flocculated biomass was clearly visible from day 7 onwards (Fig. 2e). Microscopic examination using the bright-field mode to detect the overall cell morphology and the fluorescent mode to detect autofluorescence of *Synechocystis*’s chlorophyll showed that the flocculated biomass obtained from the co-culture consisted of both *Synechocystis* and the fungi, with *Synechocystis* attached on the surface of the fungal hyphae filament (Fig. 2f–h).

**Figure 3:**
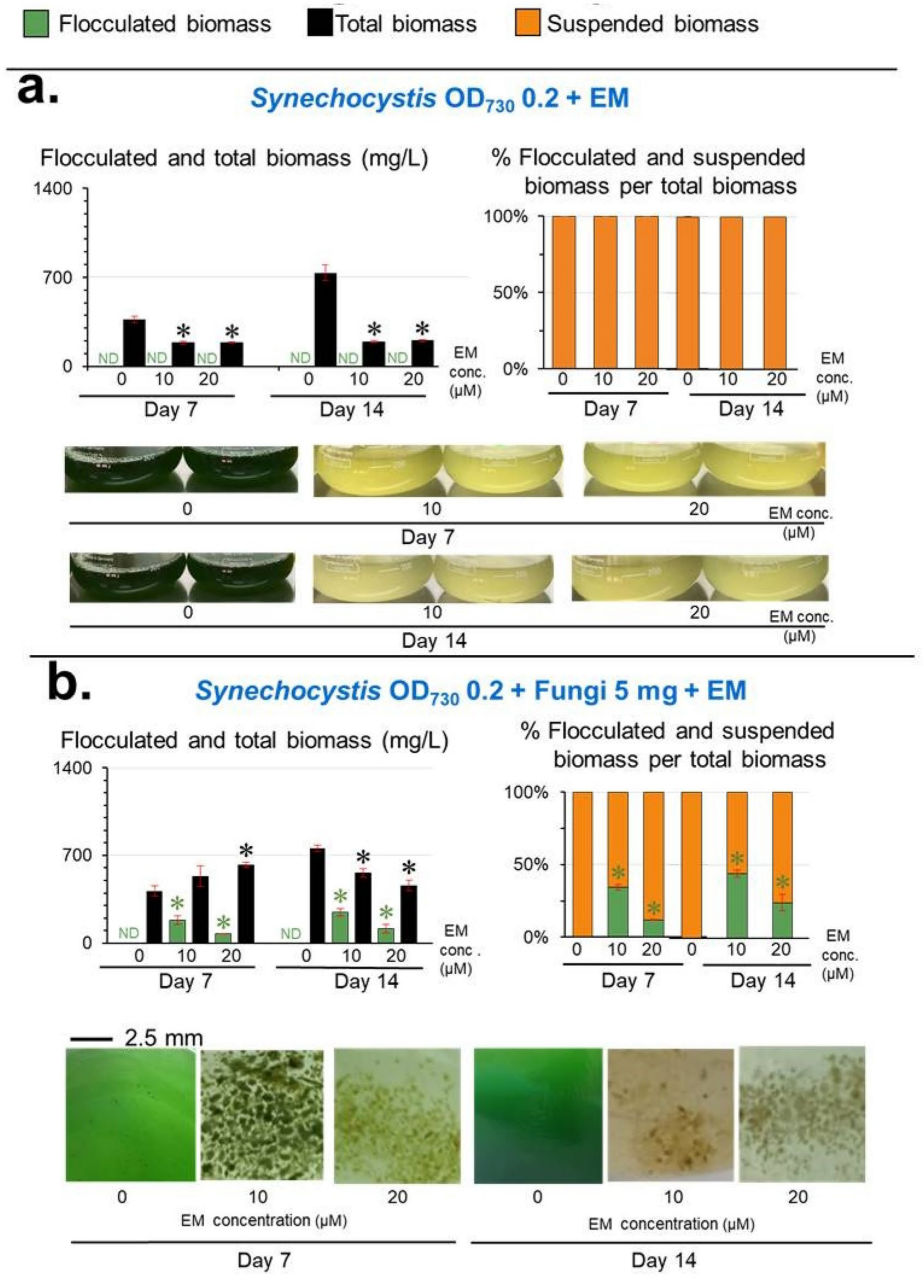
Effect of the EM concentration on the levels of *Synechocystis*-fungi flocculation. Total biomass is the sum of flocculated and suspended biomasses. Values are shown as the mean ± 1SD (n = 4). ND = not detected. (**a**) Axenic cultivation of *Synechocystis* at an initial cell density of OD_730_ = 0.2 in the presence of various EM concentrations. Representative images of the culture flasks are shown. Significantly different levels (*P < 0.05: two-tailed student’s *t*-test) between the EM-treated cells and the untreated cells at the same cultivation time and the same type of biomass. Representative images of the respective cultivation (culture flasks) are shown. (**b**) Co-cultivation between *Synechocystis* (fixed initial cell density of OD_730_ = 0.2) and the fungi (fixed initial fungi inoculation of 5 mg fresh weight/100 mL) in the presence of various EM concentrations. Significantly different levels (*P < 0.05: two-tailed student’s *t*-test) between the EM-treated cells and the untreated cells at the same cultivation time and the same type of biomass. Representative images showing cell flocculation from the respective cultivation are shown.

**Figure 4:**
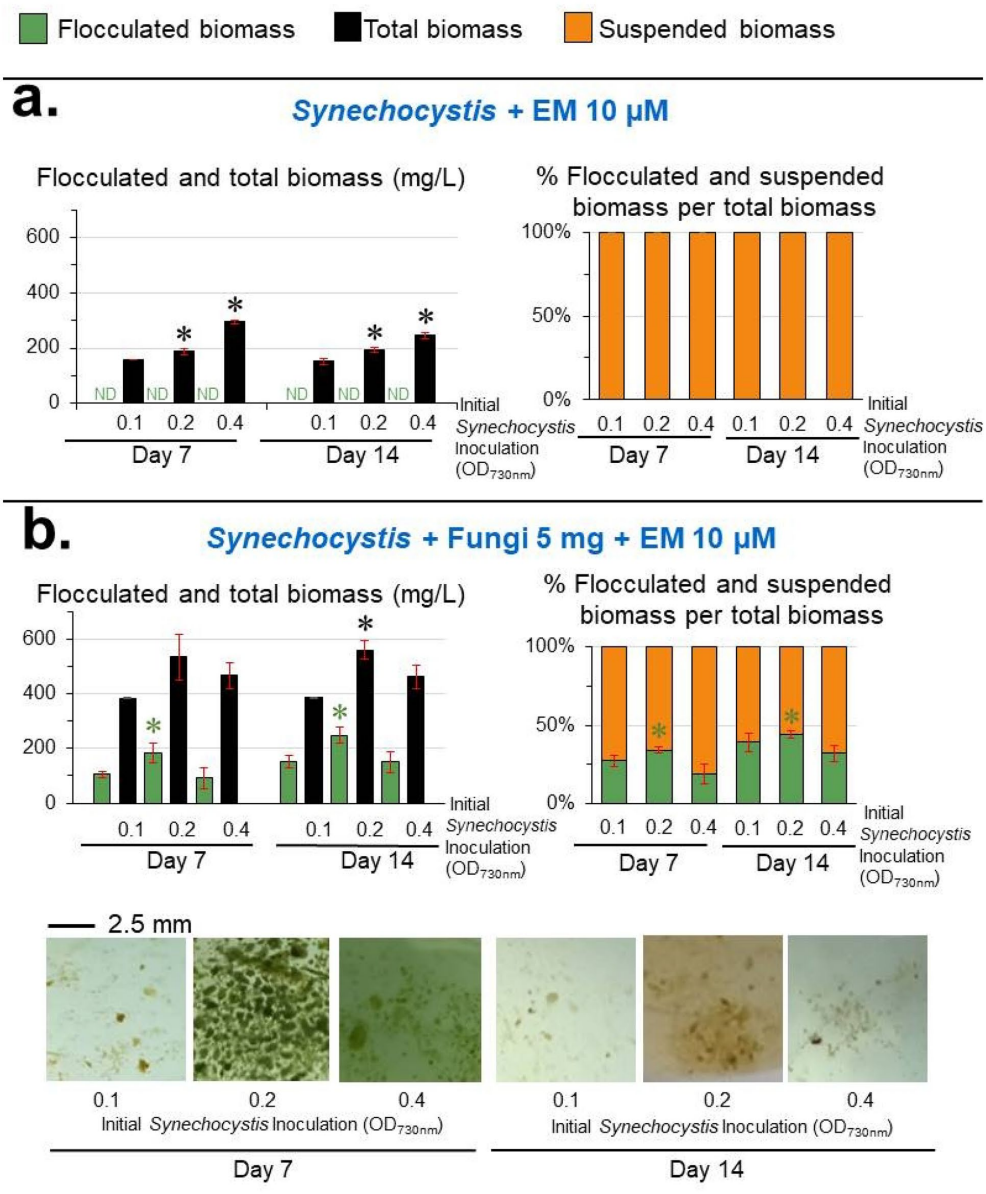
Effect of initial *Synechocystis* density on the levels of *Synechocystis*-fungi flocculation. Total biomass is the sum of flocculated and suspended biomasses. Values are shown as the mean ± 1SD (n = 4), and ND = not detected. (**a**) Axenic cultivation of various initial *Synechocystis* densities in the presence of 10 µM EM. Significantly different levels (*P < 0.05: two-tailed student’s *t*-test), compared to that of the initial *Synechocystis* inoculation at OD_730nm_ = 0.1 of the same cultivation time and the same type of biomass. (**b**) Co-cultivation between various *Synechocystis* densities and the fungi (fixed initial fungi inoculation of 5 mg fresh weight/100 mL) in the presence of 10 µM EM. Significantly different levels (*P < 0.05: two-tailed student’s *t*-test) compared to that of the initial *Synechocystis* inoculation at OD_730nm_ = 0.1 at the same cultivation time and the same type of biomass. Representative images of the cell flocculation from the respective cultivation are shown.

**Figure 5:**
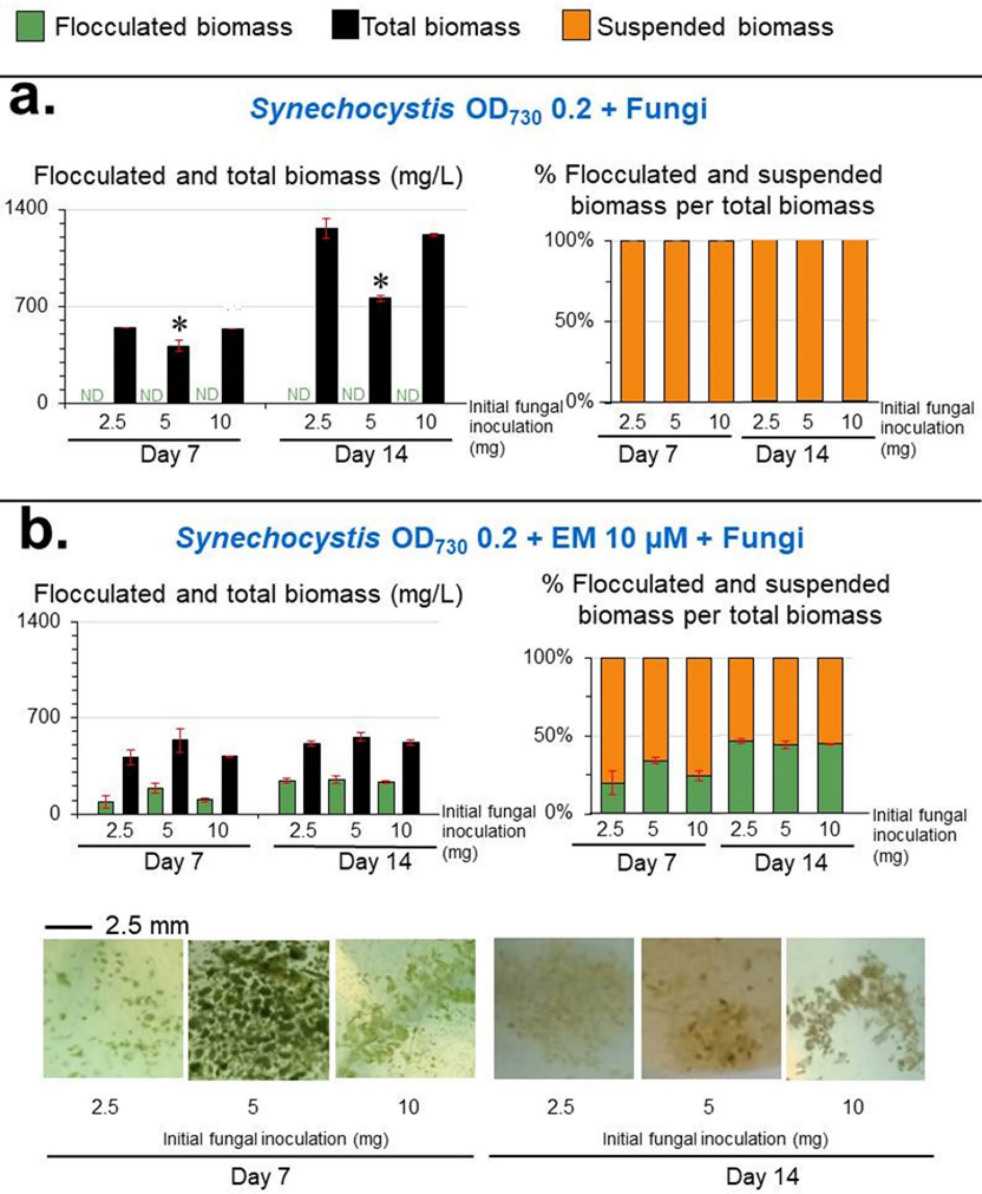
Effect of initial fungi amount on the levels of *Synechocystis*-fungi flocculation. Total biomass is the sum of flocculated and suspended biomasses. Values are shown as the mean ± 1SD (n = 4), and ND = not detected. (**a**) Co-cultivation of *Synechocystis* at an initial cell density of OD_730_ = 0.2 without EM and with the initial fungal inoculation at 2.5, 5, and 10 mg fresh weight/100 mL. Significantly different levels (*P < 0.05: two-tailed student’s *t*-test), compared to that of the initial fungal inoculation at 2.5 mg/100 mL of the same cultivation time and the same type of biomass. (**b**) Co-cultivation of *Synechocystis* (fixed initial density of OD_730_ = 0.2) and initial fungal inoculation at 2.5, 5, and 10 mg fresh weight/100 mL with 10 µM EM. Significantly different levels (*P < 0.05: two-tailed student’s *t*-test) compared to that of the initial fungal inoculation at 2.5 mg/100 mL at the same cultivation time and the same type of biomass. Representative images of the cell flocculation from the respective culture are shown.

Additionally, the co-cultivation of *Synechocystis* and the fungi without EM (Figs. 3b and 5a), and the cultivation of *Synechocystis* with EM but without the fungi (Figs. 3a and 4a), showed no cell flocculation. Thus, the cell flocculation required the presence of *Synechocystis*, fungi, and EM. It is noted that under 10–20 µM EM, the co-cultivation of both *Synechocystis* and fungi yielded higher levels of total harvested biomass than that obtained from the cultivation of only *Synechocystis* without fungi (Fig. 3a,b and Fig. 4a,b).

Fungal species present in the *Synechocystis*-fungi flocculated biomass were identified following the PCR amplification of fungal ITS rDNA, followed by next-generation sequencing. The fungal population was found to consist of five species (Table 1), with the filamentous *Purpureocillium lilacinum* and *Aspergillus protuberus* making up 71% of the overall fungal population (Table 1).

**Table 1:** Percent composition of the fungi present in the *Synechocystis-*fungi flocculated biomass.

Species of fungi	Detected frequency of ITS rDNA	% Composition in fungal population
*Purpureocillium lilacinum*	94,141	40.8
*Aspergillus protuberus*	70,920	30.7
*Sarocladium implicatum*	44,794	19.4
*Candida tropicalis*	20,459	8.9
*Dipodascaceae* sp.	465	0.2

The *Synechocystis*-fungi flocculated biomass was obtained from the co-cultivation between *Synechocystis* (initial density of an OD_730_ of 0.2) and the isolated fungi (initial inoculation of 5 mg fresh weight) in the presence of 10 µM EM for seven days. Fungal identification and frequency determination were obtained from the analysis of total fungal ITS rDNA amplified from total DNA isolated from the flocculated biomass.

Optimal initial inoculum amount of EM, *Synechocystis*, and fungi for maximal *Synechocystis*-fungi flocculation.

Increasing the initial EM concentration from 10 to 20 µM significantly reduced the *Synechocystis-*fungi flocculated biomass and caused a stronger chlorotic effect on *Synechocystis* (Fig. 3b). With respect to the initial inoculum size of *Synechocystis*, a *Synechocystis* inoculum of an OD_730_ of 0.2 yielded the highest *Synechocystis-*fungi flocculated biomass (248 ± 28 mg/L) corresponding to a 44 ± 2% flocculation efficiency at day 14 (Fig. 4), compared to the other initial inoculum sizes of *Synechocystis*. Increasing or decreasing the *Synechocystis* inoculum from OD_730_ = 0.2 slightly reduced the *Synechocystis*-fungi flocculated biomass level and % flocculation efficiency (Fig. 4). For the initial fungi inoculum, an initial inoculum of 2.5, 5, and 10 mg in 100 mL cultivation yielded comparable levels of both *Synechocystis*-fungi flocculated biomass and % flocculation efficiency at day 14 (Fig. 5).

Assessment of altered gene-expression levels of *Synechocystis* in the co-flocculated biomass compared to the suspended *Synechocystis* cells.

**Figure 6:**
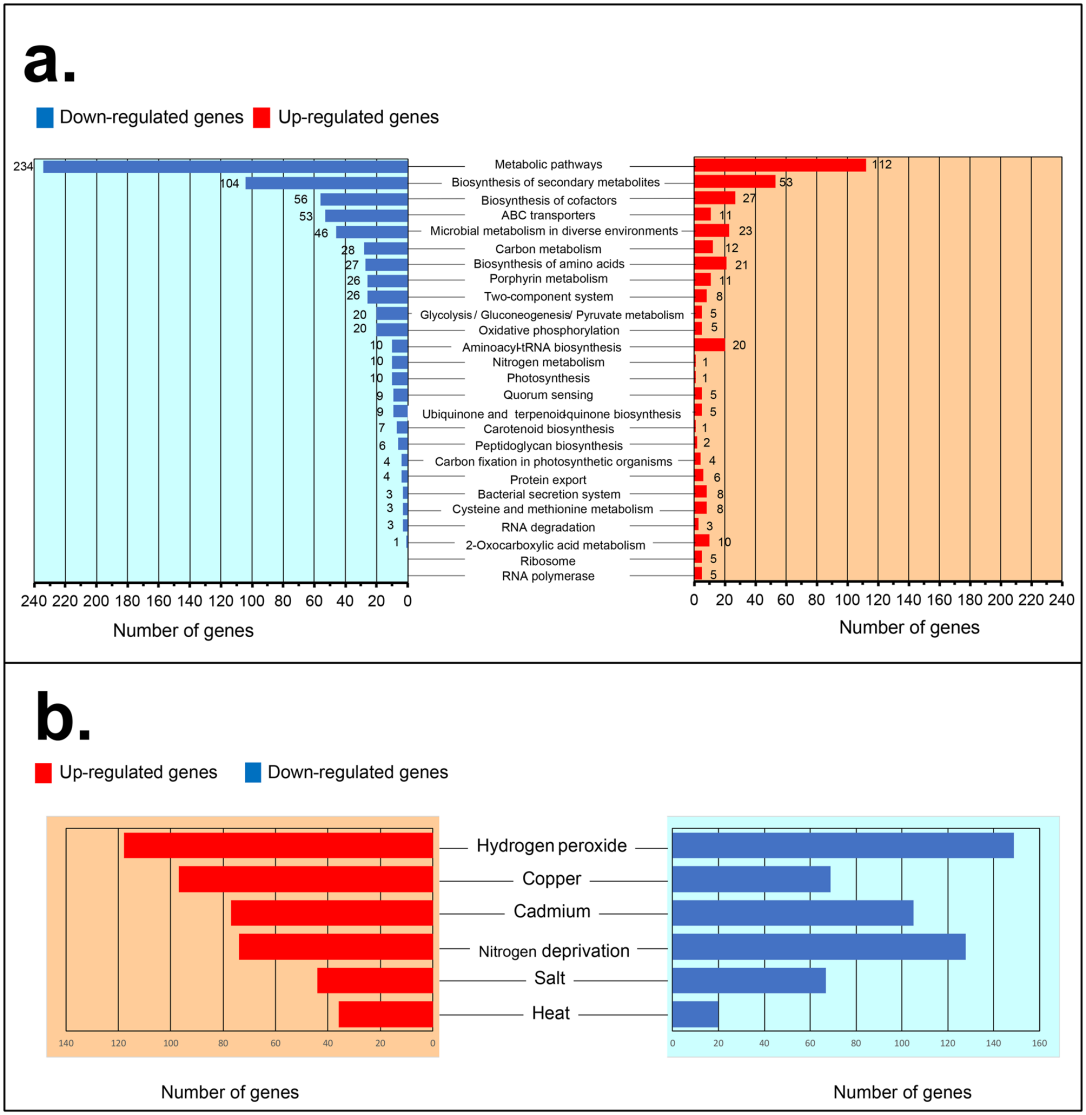
*Synechocystis* responsive genes in the flocculated cells. (**a**) *Synechocystis* responsive genes under flocculation. *Synechocystis* transcriptomics were compared between *Synechocystis* in [the *Synechocystis*-fungi flocculated biomass with EM] and [axenic suspended *Synechocystis* without EM]. The *Synechocystis*-fungi flocculated biomass was obtained from the co-cultivation between *Synechocystis* (initial density of OD_730_ = 0.2) and the fungi (initial inoculation of 5 mg fresh weight in 100 mL cultivation) in the presence of 10 µM EM. Axenic *Synechocystis* biomass was derived from the axenic culture using an initial density at OD_730_ = 0.2 without EM. The cultivation time is seven days. Data were obtained from three independent cultures. The transcriptomic data were analyzed, and the responsive genes were categorized by their cellular functions and metabolism using the Kyoto Encyclopedia of Genes and Genomes (KEGG; www.genome.jp/kegg/pathway.html)^70^. Names of the genes in each category of cellular processes are given in Supplementary Data S1. (**b**) *Synechocystis*-responsive genes under flocculation found in this study (shown in a) have also been reported to respond to other abiotic stresses: hydrogen peroxide^71^, copper^72^, cadmium^73^, nitrogen deprivation^44^, salt^74^, and heat^75^. Names of responsive genes in each stress are given in Supplementary Data S2.

Total cellular transcripts were compared between the *Synechocystis* in the *Synechocystis*-fungi flocculated biomass and the axenic *Synechocystis* suspended cells. The detected transcripts from fungi were excluded and were not used for transcriptomic analysis. Decreased transcript levels (down-regulated genes) of *Synechocystis* in the co-flocculated biomass with respect to *Synechocystis* suspended cells were found mainly in the genes responsible for metabolic pathways (234 genes), biosynthesis of secondary metabolites (104 genes) and cofactors (56 genes), ABC transporter (53 genes), and carbon metabolism (28 genes) (Fig. 6a). Thus, these cellular processes are likely to be affected by EM exposure.

Many of the responsive genes in *Synechocystis* found in the flocculated biomass in this study have also been previously reported to exhibit altered expression levels under various abiotic stresses (Fig. 6b). Of particular significance, more than two hundred responsive genes that might be involved in the flocculation in this study were also previously reported to show altered expression levels under the stress induced by hydrogen peroxide or copper treatment (Fig. 6b). Thus, these data suggested that the flocculated *Synechocystis* may encounter abiotic stress mediated by EM treatment.

*Synechocystis* responsive genes that are potentially involved in *Synechocystis*-fungi flocculation.

Total transcripts were compared between the *Synechocystis* in [the *Synechocystis*-fungi flocculated biomass with EM exposure] and [the axenic *Synechocystis* suspended cells without EM]. The twenty-one *Synechocystis* genes that showed altered transcript levels in response to the co-flocculation in this study have been previously reported to be involved in the auto-flocculation or biofilm formation of *Synechocystis* (Table 2).

**Table 2:** *Synechocystis* responsive genes potentially involved in cell flocculation that were found in both this study and in previous reports.

Gene ID	Gene product	Log_2_-fold change of expression in this study^a^			Gene expression status in the flocculated *Synechocystis* reported in previous studies^b^
		[C + F + E] compared with [C]	[C + F + E] compared with [C + E]	[C + E] compared with [C]	
*slr1674*	Hypothetical protein	7.3	1.0	6.3	Up-regulation in flocculated wild type, compared to suspended wild type^60^
*slr2076 (groEL1)*	60 kD Chaperonin 1	7.3	1.4	5.9	Normal expression in flocculated wild type, compared to down-regulation in suspended in *kpsM*-deleted strain^67^
*slr0967*	Hypothetical protein	6.9	NS	6.7	Up-regulation in flocculated wild type, compared to suspended wild type^60^
*sll0416 (groEL2)*	60 kD Chaperonin 2	6.5	1.6	4.9	Normal expression in flocculated wild type, compared to down-regulation in suspended in *kpsM*-deleted strain^67^
*slr1704*	Hypothetical S-layer protein	6.3	NS	5.4	Normal expression in flocculated wild type, compared to down-regulation in suspended *sigF*-deleted strain^68^
*sll1867 (psbA3)*	Photosystem II D1 protein	6.3	NS	6.0	Normal expression in flocculated wild type, compared to down-regulation in suspended in *kpsM*-deleted strain^67^
*slr1738*	PerR transcriptional regulator of Fur family	6.1	NS	5.8	Normal expression in flocculated wild type, compared to down-regulation in suspended in *kpsM*-deleted strain^67^
*slr1915*	Hypothetical protein	5.8	NS	5.4	Up-regulation in flocculated wild type, compared to suspended wild type^60^
*sll1823 (purA)*	Adenylosuccinate synthetase	5.5	2.0	3.5	Normal expression in flocculated wild type, compared to down-regulation in suspended in *kpsM*-deleted strain^67^
*ssr0692*	Key regulator of arginine synthesis	5.2	− 1.5	6.6	Up-regulation in flocculated wild type, compared to suspended wild type^60^
*slr0923 (wzc)*	EPS polymerization, assembly, and export	4.6	NS	4.0	Normal expression in flocculated wild type, compared to no expression in suspended *slr0923*-deleted strain^25^
*ssr3341 (hfq)*	RNA chaperone regulating pilin formation	2.6	NS	3.4	Normal expression in flocculated wild type, compared to no expression in suspended *ssr3341*-deleted strain^27^
*slr0322 (hik43)*	CheA-like protein regulating pilin biogenesis	2.0	NS	1.5	Up-regulation in flocculated wild type under salt stress, compared to suspended wild type under normal culture condition^61^
*sll1951 (slyr)*	S-layer protein on cell surface	1.9	NS	2.7	Normal expression in flocculated wild type, compared to no expression in suspended *sll1951*-deleted strain^25^
*sll1965*	Hypothetical protein in pilin regulon	1.7	NS	2.0	Up-regulation in flocculated *hik43*-overexpressed strain, compared to suspended wild type^61^
*slr0073 (hik36)*	Type IV pili sensor histidine kinase regulating pilin biogenesis	1.7	NS	1.3	Normal expression was detected in flocculated wild type under salt stress. This gene was involved in signal transduction of cell flocculation^61^
*sll0821 (cph2)*	Protein involved in inhibition of phototactic motility under blue light	1.5	NS	1.1	Up-regulation in flocculated wild type under blue light, compared to suspended wild type under normal light^69^
*slr2019*	Hypothetical protein in minor pilin regulon	− 1.2	NS	− 2.1	No expression in flocculated *slr2019*-deleted strain, compared to normal expression in suspended wild type^21,69^
*slr2017*	Minor pilin protein PilA11	− 1.6	NS	− 1.3	No expression in flocculated *slr2017*-deleted strain, compared to normal expression in suspended wild type^21,69^
*slr2018*	Minor pilin protein PilA12	− 3.6	− 1.8	− 1.8	No expression in flocculated *slr2018*-deleted strain, compared to normal expression in suspended wild type^21,69^
*slr2016*	Minor pilin protein PilA10	− 5.1	− 3.7	− 1.4	No expression in flocculated *slr2016*-deleted strain, compared to normal expression in suspended wild type^21,69^

^a^*Synechocystis* gene expression levels were obtained from the transcriptomic analysis by comparing between the two different experimental groups: [C + F + E], *Synechocystis* in the *Synechocystis*-fungi flocculated biomass cultured with EM and with the fungi; [C + E], axenic *Synechocystis* suspended cells with EM and without the fungi; [C] axenic *Synechocystis* suspended cells without EM and without Fungi. NS, not significantly different level.

^b^Gene expression status was compared between those from the *Synechocystis* flocculated cells and the *Synechocystis* suspended cells, as described in the indicated previous reports.

Interestingly, the altered expression levels of all these 21 genes were mainly caused by the EM exposure as evidenced by the transcriptomic comparison between the *Synechocystis* in [the axenic *Synechocystis* suspended cells with EM exposure] and [the axenic *Synechocystis* suspended cells without EM] (Table 2). The presence of the fungi slightly altered expression levels of only the 7 out of those 21 genes as shown by the transcriptomic comparison between the *Synechocystis* in [the *Synechocystis*-fungi flocculated biomass with EM] and [the axenic *Synechocystis* suspended cells with EM] (Table 2).

Overall, the results showed that altering expression of these 21 genes that are potentially involved in the flocculation were mainly induced by the EM exposure. Of particular importance, the up-regulated genes include the genes involved in pilin formation (*slr0322* and *slr0073*), the gene for EPS polymerization and export (*slr0923*), the genes for S-layer protein (*slr1704*, and *sll1951*), and the genes for the regulatory/signal transduction proteins (*slr1738*, *sll1823*, *ssr062*, and *sll0821*) (Table 2). Additionally, the down-regulated genes are the three genes for the minor-pilin proteins (*slr2016*, *slr2017*, and *slr2018*).

### ***Synechocystis***-fungi flocculation is also mediated by CuSO_4_ stress

**Figure 7:**
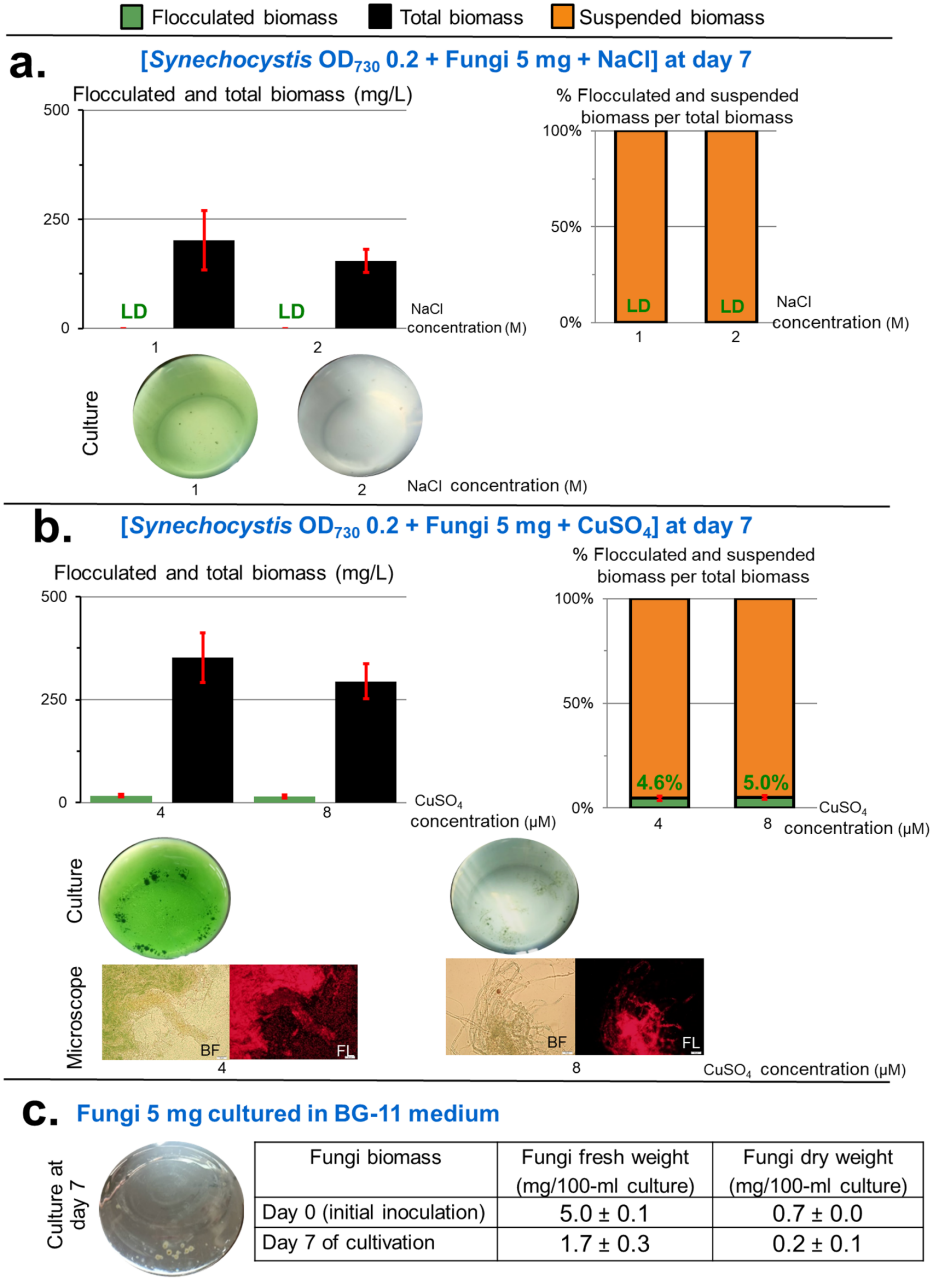
Effect of the NaCl stress and CuSO_4_ stress on the *Synechocystis*-fungi flocculation. Total biomass is the sum of flocculated and suspended biomasses. Values are shown as the mean ± 1SD (n = 4). LD: low detection level at < 2 mg/L of the flocculated biomass or < 0.5% of percent flocculated biomass per total biomass. (**a**) Effect of the NaCl stress. Co-cultivation between *Synechocystis* (fixed initial cell density of OD_730_ = 0.2) and the fungi (fixed initial fungi inoculation of 5 mg fresh weight/100 mL) in the presence of NaCl at 1 or 2 M for 7 days. Culture: representative images of the culture flasks. (**b**) Effect of the CuSO_4_ stress. Co-cultivation between *Synechocystis* (fixed initial cell density of OD_730_ = 0.2) and the fungi (fixed initial fungi inoculation of 5 mg fresh weight/100 mL) in the presence of CuSO_4_ at 4 or 8 µM for 7 days. Culture: representative images of the culture flasks. Microscope: *Synechocystis*-fungi flocculated biomass as seen by bright-field microscopy (BF, left) and fluorescent microscopy (FL, right) showing the red light resulting from auto-fluorescence of *Synechocystis* chlorophyll. (**c**) The cultivation of the fungi (using the initial inoculation of 5 mg fresh weight/100 mL) in the BG11 medium without *Synechocystis* and without EM. The fresh weights of the fungi were dried to determine the dry weight.

The presence of NaCl at 1–2 M^45^ and the presence of CuSO_4_ at 4–8 µM^46^ that have been shown to effectively induce abiotic stress in *Synechocystis*, were examined for their ability in mediating the co-flocculation between the fungi and *Synechocystis*. Interestingly, the presence of 4 and 8 µM CuSO_4_ can induce the *Synechocystis*-fungi flocculation, but with a low flocculation efficiency at 4.6–5.0% of the total biomass (Fig. 7b). The presence of 1 and 2 M NaCl results in only a trace amount of the flocculated biomass (< 0.5% of the flocculation efficiency) (Fig. 7a).

## Discussion

Cyanobacterium-fungi flocculation in liquid culture has been previously reported as follows:

*Synechocystis*-*A. oryzae*^19^, *Nostoc* sp.-*Aspergillus* sp.^20^, *Synechocystis*-*A. fumigatus*^21^, a consortium of four cyanobacterial species and *A. niger*^22^, *Microcystis aeruginosa-A. oryzae*^47^, and *Chroococcus* sp.*-A. lentulus*^48^. However, all these reported algal-fungi flocculation required an organic carbon supply in the culture medium for fungal growth or the inoculation of a substantial amount of fungal pellet without the presence of a toxic chemical. In this study, we found that the fungi can co-flocculate with *Synechocystis* in the medium without an organic carbon source, but with the presence of 10–20 µM EM (Figs. 2, 3, 4, 5) exhibiting bactericidal activity against *Synechocystis*^40^*.* Thus, *Synechocystis* may provide organic nutrients for fungal growth and EM is the primary mediator for *Synechocystis*-fungi flocculation.

Previous studies reported that co-flocculation between the eukaryotic algae *Chlorella vulgaris* and *A. oryzae* occurred when they encountered toxic arsenic. This co-flocculation helped to remove 27% of the 1.3 µM arsenic in the culture medium^49^. Additionally, co-flocculation between *Chlorella vulgaris* and *A. flavus* reduced up to 98% of 1.6 mM Cu(II)^50^. Thus, these data suggested that the formation of an algae-fungi flocculate decreased the exposure to the toxic compounds in their environment. This study revealed that in the presence of 10 µM EM, the *Synechocystis* in the *Synechocystis*-fungi flocculation survived, as evidenced by their normal blue-green color (Figs. 2e–h, 3b). However, the axenic-culture *Synechocystis* cells under the same EM concentrations showed obvious chlorotic phenotypes (Fig. 3a) and have been shown previously to have a significantly reduced cell viability^40^. Thus, the formation of *Synechocystis*-fungi flocculation protected *Synechocystis* against chlorosis. We speculated that this might be due to the reduced EM level in the culture medium caused by the co-flocculation or the co-flocculation formation helps protect *Synechocystis* against EM. Note that the actual EM concentration in the liquid media was not determined in this study (such as from the inhibition zone assay of *Escherichia coli* toward EM using the liquid-culture samples or by HPLC analysis) due to the subtle EM concentrations used in this study (0–10 µM equivalent to 0–7.3 µg/mL), which are below the minimum inhibitory concentration of EM for *E. coli* at 20 µg/mL^51^ and the lowest HPLC sensitivity at 1000–7000 µg/mL^52,53^. A more sensitive method, such as ELISA, might be used to determine the EM level.

It is noted that the abiotic stress by the CuSO_4_ exposure can also induce the *Synechocystis*-fungi flocculation but with the lower flocculation efficiently than that of EM (Fig. 7b). The cultivation of only the fungi in the BG11 medium showed that the fungi did not grow after 7 days of cultivation (Fig. 7c).

It has been reported in the co-flocculation between *Chlorella pyrenoidosa* and *A. oryzae* in liquid cultivation containing starch that these two species exchange nutrients. The *A. oryzae* secreted CO_2_ and enzymes to digest starch and protein into simple sugars and amino acids, which can be subsequently utilized by *C. pyrenoidosa*, while *C. pyrenoidosa* generated O_2_ for *A. oryzae* respiration^23^. In symbiotic lichens, algae, and fungi also exchange various nutrients for co-benefits of their metabolism^54,55^. Thus, one major known cause for these symbioses and co-flocculation between algae and fungi is the advantages of nutrient exchanges. In this study, *Synechocystis* and the fungi were cultivated in BG11 medium without organic compounds and the co-flocculation was formed in the presence of EM but not in the absence of EM (Fig. 3b). Thus, these results showed a novel finding that the primary mediating factor in the *Synechocystis*-fungi co-flocculation is the presence of the antibiotic EM, where the *Synechocystis* that flocculate with the fungi survive better (Fig. 2e–h). To minimize the bactericidal effect of EM, lower concentrations or short-term treatment of EM are needed to be tested for their efficiency in promoting the bioflocculation.

The transcriptomic analysis revealed the altered expression levels of 21 genes of *Synechocystis* that are potentially involved in the *Synechocystis*-fungi flocculation (Table 2). The altered expression of these 21 genes was mainly induced by the EM exposure (Table 2). In addition, the *Synechocystis* axenic culture treated with EM did not form *Synechocystis* flocculation (Fig. 3A). Thus, the altered expressions of the 21 genes did not cause *Synechocystis*-self flocculation, but rather contribute to the attachment between *Synechocystis* cell surface and the fungi filaments as seen under the microscope (Fig. 2g,h).

The transcriptomic comparison also showed that the presence of the fungi slightly affected the expression levels of the 7 out of the 21 genes (Table 2), indicating that the presence of the fungi was not a major cause for altering these gene expressions. These data are consistent with the results that the co-culture between *Synechocystis* and the fungi without EM did not lead to the *Synechocystis*-fungi flocculation (Fig. 5A).

For the known mechanism of cell flocculation in bacteria and cyanobacteria, cell surface molecules, such as EPS, S-layer protein, and pilin, play crucial roles in cell–cell attachment^25–27,56–58^. For algal-fungi flocculation, previous studies described that cell attachment is mediated by the different electrostatic charges on the cell surface between microalgae and fungi^21,28^, where highly negative charges on the cyanobacterial EPS^26^ attach to positive charges of saccharides on the cell surface of fungal hyphae^21,28^. The outer membrane porin responsible for EPS polymerization and transport (encoded by *wzc*) was reported to be involved in *Synechocystis* biofilm formation^25^. This study also found that *wzc* expression was highly increased up to 4.6 log_2_-fold (24-fold) in *Synechocystis* flocculated with the fungi (Table 2), suggesting EPS transport might be involved in the co-flocculation. However, all the other known EPS biosynthetic genes were not up-regulated in this work [Gene Expression Omnibus (GEO) repository, accession number GSE244152].

Inactivating the main S-layer protein gene *sll1951* significantly reduced *Synechocystis* biofilm formation, indicating that the S-layer protein is required in cell–cell attachment^25^. In this study, *sll1951* and the other two hypothetical S-layer protein genes *slr1704* and *slr1272* were upregulated at 1.9, 6.3, and 1.4 log_2_-fold, respectively, in the *Synechocystis* flocculated with fungi [Table 2 and Gene Expression Omnibus (GEO) repository: accession number GSE244152]. The S-layer protein Sll1951 has a negative charge in BG11 medium^59^, and so increased *sll1951* expression would increase the negative charge on the *Synechocystis* cell surface and would enhance the electrostatic attachment to the positive charges on the fungal surface. In addition, the S-layer has been demonstrated to help protect *Synechocystis* from EM exposure^59^. This data is correlated to the up-regulation of the *sll1951*, *slr1704*, and *slr1272* S-layer protein genes of the flocculated *Synechocystis* (Table 2) in the presence of EM in this study.

Bacterial type-IV pilins have been reported to play a role in cell mobility and cell adhesion^29,30^. Reduced expression of the genes for the three minor pilin proteins (*pilA10*, *pilA11*, and *pilA12*; which exist in the same operon^58,60^) resulted in shorter pilins, and subsequently increased the cell surface contact and promoted *Synechocystis* auto-flocculation^58^. In accord, the expression levels of *pil10, pil11*, and *pil12* were decreased by 1.6 to 5.1 log_2_-fold (Table 2), and *sycrp1*, which encodes for a positive transcriptional regulator for the *pilA9-pil10*-*pil11*-*pil12*-*slr2019* operon^58^, was also down-regulated in the *Synechocystis* flocculated with the fungi in this study [Gene Expression Omnibus (GEO) repository, accession number GSE244152]. In contrast to the minor pilin proteins, the presence of the chaperone protein Hfq to regulate pilin development is required for *Synechocystis* auto-flocculation^27^. This study also found that *hfq* was up-regulated by 2.6 log_2_-fold (Table 2) in the flocculated *Synechocystis*.

Hik36 and Hik43 regulatory proteins involved in pilin formation were previously reported to promote *Synechocystis* auto-flocculation under salt stress^61^. In this study, *hik36* and *hik43* were also up-regulated in *Synechocystis* flocculated with the fungi (Table 2). In addition, the proteins Cph2 (regulates the inhibition of cell motility and is involved in auto-flocculation under blue light exposure^62^), PurA (produces signaling AMP^63^), PII (regulates various metabolisms^64^), PerR (responds to peroxide stress^65^), and GroEL2 (a temperature-stress response protein^66^) were also significantly up-regulated in *Synechocystis* flocculated with fungi (Table 2). It is worth further examining whether these stress-responsive proteins are responsible for activating *Synechocystis*-fungi flocculation.

## Conclusion

This study found that EM mediates the co-flocculation between *Synechocystis* and the five species of fungi and that the co-flocculation helps *Synechocysti*s to survive EM exposure. Transcriptomic results suggested that the flocculation is mediated by the alteration of *Synechocysti*s pilin and cell surface composition, and this process might be regulated by a number of stress-responsive proteins. Thus, further identifying signal transduction pathways as well as characterizing cell surface alteration in both *Synechocystis* and the fungi are required to understand the mechanism of the co-flocculation. A methodology for determining biomass proportion between the cyanobacteria and the fungi in the flocculated biomass has to be established. Approaches to minimizing the fungal population and maximizing the cyanobacterial population in the flocculated biomass are also required. These approaches might be screening for optimal medium composition, as well as searching for other flocculation-stimulating compounds (especially those not affecting cyanobacterial growth) and other cyanobacterial/fungal species to achieve the maximal bioflocculation. Alternatively, an antifungal compound at a suitable concentration might be used to limit growth of the fungi. The bioflocculation with no requirement of organic compound reported in this study might be applicable for other photosynthetic microalgae and other fungal species as a novel approach for cell harvest and bioscaffold formation for CO_2_ capture.

### Supplementary Information

Supplementary Information 1.

Supplementary Information 2.

## Data Availability

The transcriptomic datasets generated and analysed during the current study are available in the Gene Expression Omnibus (GEO) repository, accession numbers: GSE244152, GSE256451, and GSE256452.
